# ONLINE EXERCISE CLASS EXPERIENCE AMONG OLDER ADULTS' MEMBERS OF COMMUNITY CENTERS IN HONG KONG DURING COVID-19

**DOI:** 10.1093/geroni/igac059.1084

**Published:** 2022-12-20

**Authors:** Janet Lok Chun Lee, Vivian Wei Qun Lou, Rick Kwan

**Affiliations:** The Hong Kong Polytechnic University, Hong Kong, Hong Kong; The University of Hong Kong, Hong Kong, Hong Kong, Hong Kong; Tung Wah College, Hong Kong, Hong Kong

## Abstract

Due to the social distancing measures, community-centres in Hong Kong has been converting exercise classes to online delivery mode through video-conferencing software since the outbreak of COVID-19. The phenomenon is new, and no research has been done to investigate older adults’ experience on it. This study adopted a descriptive qualitative methodologically orientation to understand the phenomenon. Twenty-three older adults (aged 55-89 years), with experience of participating online exercise class since COVID-19 participated in the study. Six major themes regarding their experiences emerged. Regarding positive experiences, older adults experienced convenience, increased exercise regularity, technical transformation and motivation in this new form of home-based exercise. At the same time, they experienced specific technical barriers and compensated supervision quality from the instructor. The findings of the study gave important future research direction and implications to the development of smart aging in community centres in Hong Kong.

